# Selective screening for inherited metabolic disorders in a tertiary care hospital of Karachi – A retrospective chart review

**DOI:** 10.12669/pjms.40.2(ICON).8985

**Published:** 2024-01

**Authors:** Fatima Kanani, Saba Shahid, Dua Sameer, Sidra Maqsood

**Affiliations:** 1 Saba Shahid, Chemical Pathology Section, Department of Pathology, Indus Hospital & Health Network, Karachi, Pakistan; 2 Fatima Kanani, Department of Pediatrics, Indus Hospital & Health Network, Karachi, Pakistan; 3 Dua Sameer, Department of Research, Indus Hospital & Health Network, Karachi, Pakistan; 4 Sidra Maqsood, Department of Research, Indus Hospital & Health Network, Karachi, Pakistan

**Keywords:** Children, Inherited Metabolic Disorder, Selective Screening, Tandem Mass Spectrometry

## Abstract

**Background & Objective:**

Selective high-risk screening of children suspected of having inherited metabolic disorders was conducted jointly by Chemical Pathology section and the Pediatric Department of Indus Hospital and Health Network- (IHHN) from October 2020-March 2022. Tandem mass spectrometry (MS) for newborn screening was recently introduced in a local laboratory. We did a selective high screening of children for metabolic disorders by using MS for neonates and other relevant tests for older children in our hospital. The present study was undertaken to get an estimate of the number of metabolic cases screened and identified after inclusion of an extended workup.

**Methods:**

This is a retrospective chart review of children who were selectively screened for IMDs. Patients’ records with ages ranging from birth to fourteen years of age were retrieved from the electronic records department of IHHN from October 2020 to March 2022. Records were searched for demographic data, history, signs, symptoms, and lab investigations. All relevant information was recorded on a pre-designed questionnaire.

**Results:**

A total of 178 children were screened for inherited metabolic disorders. Majority of the children screened were less than one month of age 96 (54%). Consanguinity was noted in 74 (41.5%) children. Most common symptoms observed were failure to thrive in 77 children (43%), hypoglycemia in 45 children (25%), and feeding difficulty in 36 children (20%). Inherited metabolic disorders were confirmed in 12 children out of which five had congenital adrenal hyperplasia, four had cystic fibrosis and three children had congenital hypothyroidism.

**Conclusion:**

In the present study, we were able to screen several children after inclusion of an extended metabolic workup. However, confirmation of many disorders like fatty acid oxidation defects, disorders of carbohydrate metabolism, and sphingolipidosis could not be done due to lack of confirmatory tests. We recommend that confirmatory tests of these disorders be included in local labs.

## INTRODUCTION

Inherited metabolic disorders (IMDs) are genetically inherited disorders in which there is deficiency of specific enzymes or proteins, causing a block in the normal metabolic process of protein, carbohydrate or fat metabolism.^1^ IMDs can present with various clinical signs in children ranging from vomiting, convulsions, hypoglycemia, respiratory distress, and unexplained neonatal death. In older children, IMDs can cause developmental delay, failure to thrive, and seizure disorders.^2^

In Western countries, prevalence of IMDs is 1 in 800–2500 births.^3^ In Pakistan, exact prevalence data is not available, however, multiple studies in scattered groups of selected Pakistani patients have confirmed high frequency of IMDs. Reasons stated for this include high rates of inter-marriages coupled with large family sizes.^4^

Pakistan lacks a metabolic disease registry^4^ and a national-level program for newborn screening^5^ which are due to inadequate lab facilities and high costs of screening tests.^6^ Despite these difficulties, few hospitals have started small-scale newborn screening (NBS) programs on an individual level for more common diseases such as congenital hypothyroidism.^7^

At IHHN, several children with suspected IMDs are treated in the pediatric department. Recently, newborn screening (NBS) on dried blood samples and confirmatory tests for a few metabolic disorders were initiated in a local laboratory in Karachi. NBS was done through tandem mass spectrometry (MS) for 30-40 metabolic disorders in newborns. These disorders included amino acid disorders such as arginemia, arginosuccinic acidemia, citrullinemia, hyerphenylalanemias, tyrosinemias, alkaptonuria, maple syrup urine disease, homocysteinuria, organic acidurias such as methylmalonic acidemia, propionic acidemia, isovaleric acidemia, glutaric acidemias multiple carboxylase deficiency and fatty acid oxidation defects such as medium-chain acyl-coenzyme A dehydrogenase deficiency (MCAD) and very long chain acyl- coenzyme dehydrogenase deficiency (VLCAD).

Confirmatory tests for aminoacidopathy (AA) and organic academia (OA) were done through high performance liquid chromatography (HPLC) and gas chromatography-mass spectrometry (GCMS) respectively. Recent addition of these laboratory tests offered a wider range for screening and diagnoses of inherited metabolic disorders. Therefore, during October 2020 till March 2022, we did selective high screening of children for metabolic disorders, by using MS for neonates and other relevant tests for older children. This study aimed to get an estimate of number of metabolic cases screened and identified after inclusion of extended workup, through a retrospective chart review.

### Case Definitions:

### Suspected and confirmed case of organic acidemia/aminoacidopathy/fatty acid oxidation defect

Children having history of any of the symptoms like vomiting, hypoglycemia, hypotonia, seizures, intellectual disability, feeding difficulty and failure to thrive^8^ were suspected to have AA, OA and FAO. Screening for these disorders was done through MS. Confirmation of AA and OA was done through HPCL and GCMS. Fatty acid oxidation defect (FAO) could not be confirmed due to non-availability of confirmation test.

### Suspected and confirmed case of congenital adrenal hyperplasia (CAH)

Children having history of any of the symptoms like vomiting, dehydration, electrolyte imbalances, acidosis, hypoglycemia, and ambiguous genitalia were suspected to have CAH,^9^ confirmation was accepted when serum 17 hydroxy progesterone (17-OHP) level was > 10ng/ml.^10^

### Suspected and confirmed case of congenital hypothyroidism (CH)

Children having history of any of the symptoms like prolonged jaundice, feeding difficulties, constipation, macroglossia, pedal edema, umbilical hernia, hypotonia, or goiter were suspected to have CH.^11^ Up till 15 days of life, confirmation of CH was done when TSH was >20 mU/L and FT4 was <11.8 pmol/L. After 15th day of life CH was labelled when TSH was more than 10 mU/L and FT4 was < 11.3 pmol/L.^12^

### Suspected and confirmed case of cystic fibrosis (CF)

Children having history of any of the symptoms like repeated chest infections with or without diarrhea, clubbing, and radiological findings suggestive of cystic fibrosis. Confirmation of CF was done through sweat chloride quantification with a cut off > 60 mmol/L.

### Suspected case of galactosemia, glycogen storage disease (GSD), lysosomal storage disorder and mucopolysaccharidosis (MPS)

Children were suspected to have these disorders when history and clinical examination was suggestive. Galactosemia was screened by doing urine analysis for sugar chromatography, GSD and sphingolipidosis were screened by liver/bone marrow biopsy and MPS was screened through skeletal x-rays.

## METHODS

This is a retrospective chart review of children who were selectively screened for IMDs. Patients’ records with ages ranging from birth to fourteen years of age were retrieved from the electronic records department of Indus Hospital & Health network (IHHN) from October 2020 till March 2022. Electronic records were searched using keywords, IMD, hypoglycemia, hypothyroidism, congenital adrenal hyperplasia, metabolic disorders, organic acidemia and aminoacidopathy. Only those cases were shortlisted for which complete screening and confirmatory lab investigations were present. Records were searched for demographic data, history, signs, symptoms, and lab investigations. All the relevant information was recorded on a pre-designed questionnaire.

Children with age less than two weeks were screened for aminoacidopathy, organic acidemia and fatty oxidation defect through extended metabolic workup done through tandem mass spectrometry on dried blood spots.

Confirmation of aminoacidopathy was done via HPLC on plasma specimens and organic acidemia was confirmed by GCMS on urine. CH screening was done through serum TSH analysis, while confirmation was done by free thyroxine (FT4); both by chemiluminescence technique. Cystic fibrosis was screened by conductivity and diagnosed by sweat chloride quantification by coulometry and CAH was diagnosed by 17 OH progesterone analysis on chemiluminescence. Confirmation of lipidosis, galactosemia, mucopolysaccharidosis and MCAD could not be done due to nonavailability of confirmatory tests.

### Inclusion Criteria

All children between 0-14 years of age coming to IHHN from October 2020-March 2022, with the history and examination suggestive of IMDs were included.

### Exclusion Criteria

Preterm neonates, children with sepsis and hypothyroid children who had autoimmune disease were excluded.

### Ethical consideration

The study was approved by the institutional ethical review board before commencement. Study IRB Number is: IHHN_IRB_2022_03_013

### Statistical analysis

All patient data was de-identified and replaced with study codes in the analysis stage and only research members had access to the password-protected data repository. Frequency was reported for categorical variables.

## RESULTS

Total of 178 children were screened for inherited metabolic disorders. Out of these, 101 (57%) were females. The majority of children screened were less than one month of age, 96 (54%). Consanguinity was noted in 74 (41.5%) children while consanguinity data was missing in 43(24%) children. Most common symptoms observed were failure to thrive, 77 children (43%), hypoglycemia 45 children (25%) and feeding difficulty 36 children (20%), Table-I. Screening tests analysis showed acidosis in 16 (13%), positive urinary ketones in 52(89%) and raised serum lactate in 36 (80%) of children, Table-I.

**Table-I:** Demographic characteristics and baseline investigations of study participants.

Demographic (N=178)	Frequency (n) %
***Age***	
<1 month	96 (54)
1-12 month	43 (24)
>12 month	39 (22)
***Gender***	
Female	101 (57)
Male	77 (43)
***Consanguinity***	
Positive	74 (41.5)
Negative	61 (34)
Unknown	43 (24)
Mental retardation in sibling	23 (13)
Death of sibling	43 (24)
Hypoglycemia in sibling	31 (17.4)
Unexplained fit in sibling	11 (6)
***Signs and symptoms***	
Vomiting	35 (19.6)
Hypoglycemia	45 (25)
Hypotonia	16 (9)
Seizures	09 (5)
Feeding difficulty	36 (20)
Failure to thrive	77 (43)
Coarse facies/dysmorphology	11 (6)
Developmental delay	26 (14.6)
***Investigations***	
Acidosis (120)	16 (13)
***Urinary ketones (58)***	
Positive	52 (89)
Negative	6 (10)
Raised serum lactate (45)	36 (80)
Deranged ALT (60)	24 (40)
Chest infiltrates (47)	17 (36)

Aminoacidopathy, organic acidemia and fatty acid oxidation defects were the most common disorders suspected (93) followed by congenital hypothyroidism (31), Cystic fibrosis (16) and congenital adrenal hyperplasia (15) Table-II.

**Table-II:** Details of suspected and confirmed metabolic disorders.

Metabolic disease suspected (n)	Screening tests performed	Confirmatory test performed	Final results		

			Normal n	Disorder confirmed n	Disorder remained suspected- n
OA/AA/FAO (n=93)	MS	AA: HPLC OA: GCMS	90	-	03 (FAO)
Galactosemia/GSD (n=10)	Urine for sugar chromatography Urine for reducing sugars	Not available	03	-	07
CAH (n=15)	Serum electrolytes, renin, cortisol	17-OHP	10	05	-
CF (n=16)	sweat chloride conductivity	sweat chloride coulometry	12	04	
Lysosomal storage disorder. Gaucher and Niemann-Pick disease (n=11)	Liver biopsy/ bone marrow biopsy	Not available	06	-	05
Lysosomal storage disorder Mucopolysaccharidosis (n=2)	Clinical features x-ray	Not available	-	-	02
CH (n=31)	Thyroid stimulating hormone (TSH) by chemiluminescence	FT4	28	03	-

OA= organic acidemia, AA= Aminoacidopathy, FAO=fatty acid oxidation defect, GSD= glycogen storage disease, CAH= congenital adrenal hyperplasia, CF= cystic fibrosis, CH= congenital hypothyroidism.

Total of 93 children were screened for AA, OA and FAO, out of these, 90 children were screened normal by MS while three continued to have suspicion of fatty acid oxidation defect. Ten children were screened for galactosemia/GSD, out of which seven children still had suspicion of disease that could not be confirmed. CAH and CF were confirmed in five and four children respectively. Eleven children were screened for lysosomal storage disorders through liver/bone marrow biopsy. Five children had disease specific histopathology, but diagnosis could not be confirmed due to non-availability of confirmatory tests. Out of thirty-one children screened for CH, three cases were confirmed, Table-II.

## DISCUSSION

In our study, twelve cases of different disorders were confirmed which included five cases of CAH, four cases of CF, and three cases of CH. However, seven cases of lysosomal storage disorder, and galactosemia each, and three cases of FAO remain undiagnosed; when we started selective high-risk screening of children in over a period of eighteen months by using extended metabolic workup. FAO defects were suspected in three children due to presence of acidosis, hypoglycemia, absent ketonuria, and carboxylic acid in urine.

Consanguinity has been proposed as a major risk factor for inherited disorders. In Pakistan, the overall reported rate of consanguineous marriage is between 40-60%,^13^,^14^ and we observed consanguinity in 74 (41.5%) cases.

Marriage between first-degree cousins is very popular in Pakistan due to religious, cultural, and economic reasons. In many tribes, inter-family marriages are done to keep fortunes within the same family. However, cousin marriages promote the propagation of diseased genes in future generations. Inborn errors of metabolism are monogenic disorders that are mostly inherited in autosomal recessive manner or less frequently in autosomal dominant or X-linked patterns. Vast majority of mutations responsible for IEM are small DNA changes that affect single or several nucleotides. Due to consanguinity these mutated genes are transmitted to children and result in metabolic diseases.^15^ An effective way to reduce IMDs in our population can be active counselling against intermarriages, by healthcare professionals.

In the present study, risk factors, identified in family members of suspected children, were mental retardation (13%) and hypoglycemia (17.4%). Whereas clinical features commonly seen in suspected children included failure to thrive (43%), hypoglycemia (25%), and developmental delay (14.6%). Similar findings are also reported in other studies.^2^,^16^

Generally, children with inherited metabolic disorders, present with varied and non-specific symptoms, which poses a diagnostic dilemma. In the present study, we also noted vague and diverse symptoms. In our setup, poor socioeconomic conditions predispose children to problems like malnutrition, vomiting, and infections which may mimic symptoms of inherited metabolic disorders. There is a possibility that high-risk screening may include many false positive cases if only vague clinical features are used. Therefore, we recommend screening children with clinical features specific to the disorders and including suggestive family history in high-risk screening for IMDs.

Disorders most frequently suspected on basis of clinical features and initial laboratory investigations included OA, AA, CH, CF and CAH. A meta-analysis of 18 studies conducted in Pakistan identified 536 confirmed cases of IMD other than CH and 212 cases of CH. The most frequent disorders reported in this meta-analysis were OA (n=220), carbohydrate storage disorders (n=188) and lysosomal storage disorders (n=188).^17^ Spectrum of metabolic disorders in the present study follows the same pattern as reported in the literature except we found fewer cases of carbohydrate and lysosomal storage disorders. The observed difference could be due to small sample size of the present study. A point worth noting is that although most frequent disorders reported in our population are OA, carbohydrate, and lysosomal disorders yet confirmatory test is only available for OA. Most cases of lysosomal and carbohydrate storage defects still remain undiagnosed due to lack of confirmatory tests. We suggest that efforts should be made to include confirmatory tests of common inherited disorders present in Pakistan which will not only reduce costs of outsourcing investigations but will also help in diagnosing suspected cases early.

The introduction of tandem mass spectrometry in clinical diagnostics has revolutionized the concept of newborn screening for inherited metabolic disorders. The test simultaneously screens for multiple disorders on dried blood spots (DBS). However, mass spectrometry is a screening test, and a suspected disorder still needs confirmation through a confirmatory test. As a result, the overall test cost rises and may be unaffordable for many people in Pakistan.

Pakistan is a low-middle-income country with a high perinatal mortality rate of 58.2/1000 births^18^ and maternal mortality rate of 186/100,000 live births.^19^ There are overwhelming problems in the health system which include lack of clean water, poor sanitation, presence of rampant infectious diseases, and poor access to robust health system.^20^ Additionally, many challenges in the implementation of NBS have been identified in our setup of which few are, lack of government support, large numbers of out-of-hospital births, few centers of genetic and metabolic services in Pakistan,^7^,^21^ insufficient numbers of trained medical and auxiliary staff, lack of awareness and the high cost of medications and food for special medical purposes (FSMPs).^22^

Considering these facts question arises, is it right to start newborn screening in Pakistan where healthcare is so poor? It is our opinion that solving other health problems should take precedence over introducing overarching newborn screening in Pakistan for now. There is no denial about the importance of integrating NBS for inherited metabolic disorders into the national health system. However, till the time it is implemented, we suggest that efforts are made for starting a targeted NBS panel like screening for congenital hypothyroidism.

The estimated incidence of CH is one in 1600 live births.^22^ The cost of CH screening is affordable, and treatment of the disease is cheap and available. Introduction of national screening for CH will result in saving many children with congenital hypothyroidism from permanent disability.

### Limitations

The present study had a small sample size due to which the results of our study cannot be generalized. We also did not perform serum ammonia levels routinely due to non-availability of the test in our institute. This may have resulted in insufficient screening.

## CONCLUSION

In the present study we were able to screen several children after the inclusion of extended metabolic workup. However, confirmation of many disorders like fatty acid oxidation defect, disorders of carbohydrate metabolism, and sphingolipidosis could not done due to lack of confirmatory tests. We recommend that confirmatory tests of these disorders are included in local labs.

### Authors’ Contribution:

**SS and FK** conceived and designed the study, were involved in the manuscript write-up. **DS and SM** helped in data extraction, statistical analysis, and preparation of manuscript. **SS** is responsible for the integrity of research.
